# A Meta-Analysis of Self-Administered vs Directly Observed Therapy Effect on Microbiologic Failure, Relapse, and Acquired Drug Resistance in Tuberculosis Patients

**DOI:** 10.1093/cid/cit167

**Published:** 2013-03-13

**Authors:** Jotam G. Pasipanodya, Tawanda Gumbo

**Affiliations:** Office of Global Health and Department of Medicine, The University of Texas Southwestern Medical Center, Dallas

**Keywords:** directly observed therapy, self-administered therapy, tuberculosis, acquired drug resistance, microbiologic failure

## Abstract

***Background*** Preclinical studies and Monte Carlo simulations have suggested that there is a relatively limited role of adherence in acquired drug resistance (ADR) and that very high levels of nonadherence are needed for therapy failure. We evaluated the superiority of directly observed therapy (DOT) for tuberculosis patients vs self-administered therapy (SAT) in decreasing ADR, microbiologic failure, and relapse in meta-analyses.

***Methods*** Prospective studies performed between 1965 and 2012 in which adult patients with microbiologically proven pulmonary *Mycobacterium tuberculosis* were separately assigned to either DOT or SAT as part of short-course chemotherapy were chosen. Endpoints were microbiologic failure, relapse, and ADR in patients on either DOT or SAT.

***Results*** Ten studies, 5 randomized and 5 observational, met selection criteria: 8774 patients were allocated to DOT and 3708 were allocated to SAT. For DOT vs SAT, the pooled risk difference for microbiologic failure was .0 (95% confidence interval [CI], −.01 to .01), for relapse .01 (95% CI, −.03 to .06), and for ADR 0.0 (95% CI, −0.01 to 0.01). The incidence rates for DOT vs SAT were 1.5% (95% CI, 1.3%–1.8%) vs 1.7% (95% CI, 1.2%–2.2%) for microbiologic failure, 3.7% (95% CI, 0.7%–17.6%) vs 2.3% (95% CI, 0.7%–7.2%) for relapse, and 1.5% (95% CI, 0.2%–9.90%) vs 0.9% (95% CI, 0.4%–2.3%) for ADR, respectively. There was no evidence of publication bias.

***Conclusions*** DOT was not significantly better than SAT in preventing microbiologic failure, relapse, or ADR, in evidence-based medicine. Resources should be shifted to identify other causes of poor microbiologic outcomes.

## Abstract (precis)

We performed a meta-analysis of prospective studies to compare microbiologic failure, relapse, and acquired drug resistance in tuberculosis patients on directly observed versus self-administered therapy. Directly administered therapy was no better than self-administered therapy.

Tuberculosis treatment with short-course chemotherapy has 3 aims: rapid bactericidal activity, which is measured by sputum conversion; sterilizing activity, which is measured by relapse; and suppression of acquired drug resistance (ADR). The World Health Organization's (WHO) DOTS (directly observed therapy, short-course) program was developed to ensure success of this chemotherapy. DOTS has 5 components: political commitment by governments, improved laboratory services, a continuous supply of good-quality drugs, documentation of individual patients’ success and program progress toward set targets, and direct observation by a healthcare worker of each patient swallowing pills (ie, directly observed therapy [DOT]). Historical trends of the decline of multidrug-resistant tuberculosis rates with implementation of the program, especially the dramatic reports from New York City and other large cities, provided powerful examples of the success of the program [1–4]. DOT, the namesake and heart of the program, is the most expensive [5–7]. However, DOT is considered by the World Bank to be one of the “most cost-effective of all health interventions, and indispensable to preventing ADR and relapse” [8, 9]. The several studies that were pivotal to the adoption of the DOTS program were retrospective, or employed quasi-experimental designs, and often emphasized the benefit of program-defined treatment outcomes [1–4, 8–12]. They did not tease out the effect of DOT from other program components. In contrast, one meta-analysis of prospective studies found no major benefit of DOT compared to self-administered therapy (SAT) for program-defined outcomes such as “cure” and “completion of treatment” in both active and latent tuberculosis [13]. In another systematic review, there was also no significant benefit for the outcome of recurrence [14]. However, in some high-burden countries such as in South Africa, up to 77% of recurrence is due to new infection and not relapse [15, 16].

Because DOT is now the accepted standard of care everywhere, performance of randomized controlled trials in which some patients are randomized to SAT or DOT or placebo pills to see if ADR emerges more easily would be unethical [17]. To address this limitation, we recently performed hollow-fiber studies in which various degrees of nonadherence were examined during both bactericidal and sterilizing effect [18]. Surprisingly, microbiologic failure occurred only when >60% of doses were missed, but no ADR was encountered. Thus, we hypothesize that DOT has no impact on rates of sputum conversion, ADR, or relapse in tuberculosis patients. To test that hypothesis, we performed a meta-analysis of prospective clinical studies that compared DOT to SAT and reported microbiologic outcomes. We were particularly interested in microbiologic outcomes as primary outcomes, as it is a standard tenant of infectious diseases therapeutics that the best evidence for eradication of pathogens or ADR, or relapse, is microbiologic demonstration [19], and not program factors such as “completion of therapy.”

## METHODS

### Definitions

We used WHO definitions [20]. DOT refers to the practice of supervising tuberculosis patients swallowing all their pills over the entire course of treatment by trained health personnel who are accountable to tuberculosis control staff. SAT refers to unsupervised administration of prescribed antituberculosis drugs by patients. We defined partial DOT as the practice in which patients are on DOT for only portions of the therapy duration. Defaulting refers to missing a cumulative ≥2 months of doses after initially taking at least 1 month's worth of medication. Patients reported as lost to follow-up by randomized clinical trials were included in the defaulting category. Microbiologic failure refers to positive smear microscopy or culture at the fifth month or later on therapy. Patients who had their treatment changed for persistent bacteriologic positivity or because of radiologic and/or clinical deterioration, including those with “doubtful responses,” were classified as having failed treatment. ADR was defined as new or additional resistance to 1 or more of the first-line antituberculosis drugs among failures or relapses. Relapse was when a patient was declared cured but subsequently developed microbiologically proven disease [20]. Molecular genotyping of repeat isolates was not performed.

### Search Strategy

We searched PubMed, Embase, ISI Web of Science, and the Cochrane Library for studies published between 1 January 1965 and 31 December 2012. There was no exclusion of articles by language. Bibliographies of original articles, key reviews, and consensus statements were also searched for additional relevant studies [8, 10, 13, 14]. The following Medical Subject Heading terms and strategy was used: *directly observed therapy* OR *supervised therapy* OR *directly observed treatment strategy* OR *DOT* OR *DOTS* AND *self-administered therapy* OR *self-supervised therapy* OR *unsupervised therapy* AND *tuberculosis*. In addition, we also searched for articles in the gray literature at Inside Conferences, clinicaltrials.gov, and Open Grey (System for Information on Grey Literature in Europe; http://www.opengrey.eu).

### Study Selection Criteria

Inclusion criteria were prospective studies in which patients were diagnosed by microscopic examination of sputum smear or culture and were separately assigned to either DOT or SAT, treatment using a short-course chemotherapy regimen that includes isoniazid, rifampin, and pyrazinamide and evidence of evaluation for microbiologic failure. Studies were limited to prospective data from observational studies or controlled trials with concurrent controls. We excluded retrospective studies to avoid selection and information biases, studies carried out in children, studies that used retreatment regimens, and treatment in patients with a prior history of tuberculosis.

### Data Extraction and Quality Assessment of Included Studies

Study selection was done independently by the 2 investigators. Reviewer agreements were measured using the κ statistic. The quality of each trial was graded by use of validated scores [21]. Disagreements were resolved by consensus.

### Outcomes

The primary outcome was microbiologic failure. The secondary outcomes were ADR, relapse, and default.

### Standards

We followed the Preferred Reporting Items for Systematic Reviews and Meta-Analyses guidelines [22].

### Data Analysis

We quantified heterogeneity of effect using the *I^2^* statistic [23, 24]. We calculated the incidence rate (IR) and 95% confidence intervals (CIs) for DOT or SAT, for each study for each of the outcomes based on the number of events reported in each original study. We also computed for a second effect size measure, which is the risk difference (RD). This was used because several cells had zero outcomes events, which makes it difficult to calculate relative risk (RR) without imputation of data or excluding studies. However, all 3 effect sizes were reported, with IR and RD considered the primary. To permit unbiased comparison of outcome, we employed an “intention to treat” strategy (ie, by original assigned treatment groups, irrespective of whether treatment was subsequently changed), except when not stated by the primary study, when we analyzed outcomes as all patients randomized [24]. We decided a priori to use the DerSimonian and Laird random methods to pool effect size across studies, as these methods would provide more conservative CIs [23, 25, 26]. Fixed-effects models were used to pool effect size if there was no significant heterogeneity (ie, *I^2^* ≤ 50%); otherwise, mixed-effects models were used for *I^2^* > 50%. We employed mixed-effects models, in which random-effects analyses were used to combine IR of groups within each study, using Comprehensive Meta-Analysis software (Biostat Inc, Englewood, New Jersey). Study-to-study variance (*T^2^)* was not pooled across studies; however, it was computed within groups and was not assumed to be the same for all groups. Publication bias and small study effects were systematically evaluated by visual inspection for funnel plot asymmetry and by use of the Egger test [23, 26].

### Subgroup and Sensitivity Analysis

First, we examined the effect of removing one study at a time on effect size for microbiologic failure, ADR, and relapse. Second, we examined the effect of study design (randomized controlled trials vs observational studies) on effect size. Third, we examined whether combining all patients classified as partial DOT with either DOT or SAT led to significant changes in effect size. Fourth, we examined the role of study locale (rural patients vs urban patients) on effect size. Fifth, we examined the effect of study quality score on effect size.

### Meta-Regression Analysis

To further explore potential source of heterogeneity, we performed meta-regression analyses in which study design and study locale were simultaneously examined as covariates. Random-effects meta-regression was utilized; we expected some unexplained or “residual” heterogeneity. The weight for each trial was equal to the inverse of the sum of the within-trial variance and the residual between-trial variance, in order to correspond to a random-effects analysis. An iterative method providing restricted maximum likelihood estimates of regression parameters, their asymptotic variance, and the residual heterogeneity variance was performed in Stata version 12.

## RESULTS

### Study Selection and Characteristics of Included Studies

**Figure 1.**
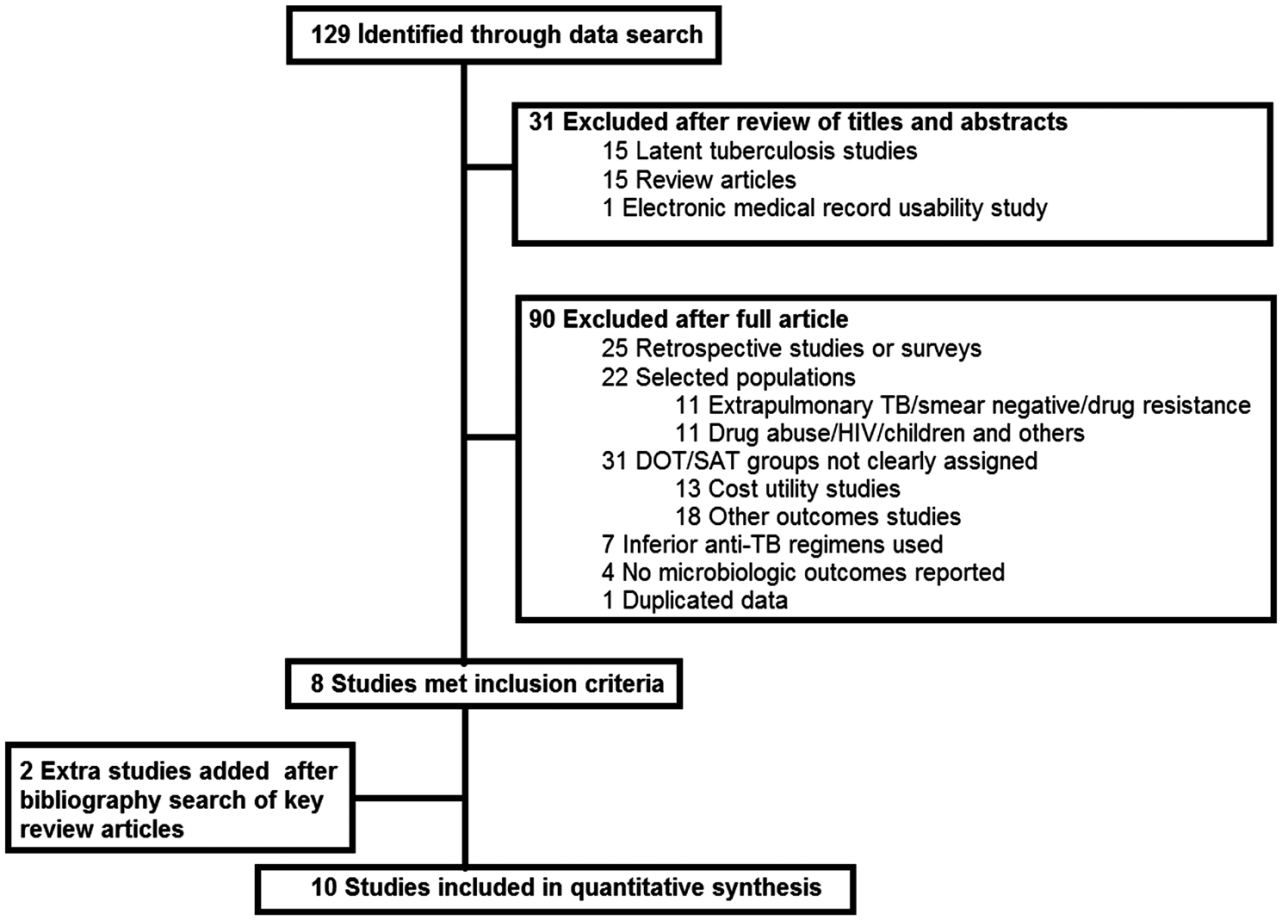
Summary of literature search and study selection for the meta-analysis. Abbreviations: DOT, directly observed therapy; HIV, human immunodeficiency virus; SAT, self-administered therapy; TB, tuberculosis.

Ten of 129 initially identified studies (8%) met selection criteria [5, 27–35], as shown in Figure 1. The *κ* value was 0.92 for the inclusion of studies and 0.90 for the rating of trials on considered methodologic aspects. There were 5 randomized controlled trials and 5 observational studies. The characteristics of included studies are shown in Table 1, as is the quality score for each study, which demonstrates that all 10 were good-quality studies. The combined number of participants enrolled in the selected studies was 13 752. From these, 13 112 (95%) participants were assigned or randomized to intervention: 8774 (67%) to DOT, 630 (5%) to partial DOT, and 3708 (28%) to SAT. Thus, the proportion of patients who received partial DOT was small, and this group was excluded from further computation of effect size.

**Table 1.** Characteristics of 10 Studies Selected for the Meta-Analysis

Study Reference	Place (Study Period)	Type of Location	Regimens Examined^a^	Patients Assigned to Interventions	Study Quality	Patients Selected	Intervention Procedures	
							DOT	SAT
Randomized trials								
[5]	Pakistan (1996–1998)	Rural/urban	2HRZE_7_/6HE_7_	497	4	New, sputum positive, >15 y	HCW at facility monitored 6×/wk; trained CM and FM monitored monthly during collection of antituberculosis drugs	Twice-monthly review and to collect antituberculosis drugs
[32]	Cape Town, South Africa (1994–1995)	Urban	2HRZ_7_/4HR_7_; 3HRZE_7_/6HRE_7_	216	4	New and retreatment, drug susceptible, >15 y	HCW monitored DOT at clinic during working hours, 5×/wk for IP, then thrice weekly for CP for new patients	Patient self-supervised, nurse reviewed adherence card weekly during clinic visit to obtain antituberculosis drugs
[33]	Cape Town, South Africa (1994–1995)	Urban	2EHRZ_7_/6EH_7_; 2EHRZ_2_/4EHR_2_; 2HRZ_2_/4HR_2_	156	4	New and retreatment, drug susceptible, >15 y	HCW at clinic and trained LHW. Patients on LHW supervision took meds several times/wk at LHW home	Patient self-supervised, nurse reviewed adherence card weekly during clinic visit to obtain antituberculosis meds
[34]	Madras and Chennai, India	Urban	2EHRZ_7_/6EH_7_; 2EHRZ_2_/4EHR; 2HRZ_2_/4HR_2_	1203	3	Sputum smear positive, >15 y.	HCW at clinic at least once/wk	Completely unsupervised, weekly drug collection during IP and twice monthly during CP
[35]	Thailand (1996–1997)	Rural/urban	2HRZE_7_/4HR_7_	837	4	New, sputum positive, >15 y	CM, FM, both trained and monitored twice/mo during IP and once/mo in CP; compliance monitored by use of treatment cards, pill counts and urine test for rifampin. HCW; monitored daily	One-mo supply of drugs after diagnosis and after follow-up visits. No supervision
Observational studies								
[27]	Blackburn, UK (1988–2000)	Urban	2HRZ_3_/4HR_3_; 3HRZE_3_/6HRE_3_	205	3	Sputum smear positive	HCW, at clinic thrice weekly. DOT mandatory for noncompliant or at-risk patients	Monthly review, random urine testing and pill counts: all received FDC
[28]	Southern Thailand (1999)	Rural/urban	2HRZE_7_/4HR_7_; 2HRZE_3_/4HR_3_	411	4	New, smear positive	DOT supervisors not stated; various levels of DOT examined. Strict DOT referred to observers actually watching patients swallow all the drugs during the first 2 mo	Not strict DOT, referred to as SAT
[29]	San Francisco, USA (1998–2000)	Urban	2HRZE_3_/4HR_3_	372	3	Culture positive	HCW at clinic, home, or workplace; enablers given: DOT mandatory for at-risk patients and noncompliance	Monthly review
[30]	Bangkok, Thailand (2004–2005)	Urban	2HRZE_7_/4HR_7_	1256	4	New, smear positive	Center-based (HCW), family-based (FM), or hybrids of center/SAT based; or center + family DOT	Patients who could not attend to be accommodated in center- or family based DOT, were assigned SAT
[31]	Thailand (2004–2006)	Rural/urban	2HRZE_7_/4HR_7_; Other	8031 (only 7070 analyzed for end-of- treatment analysis)	4	New, smear positive, drug susceptible	HCW observed ingest antituberculosis drugs at least 5×/week, trained FM observed patient ingest antituberculosis and record event	Self-administered antituberculosis, reviewed monthly.

**Figure 2.**
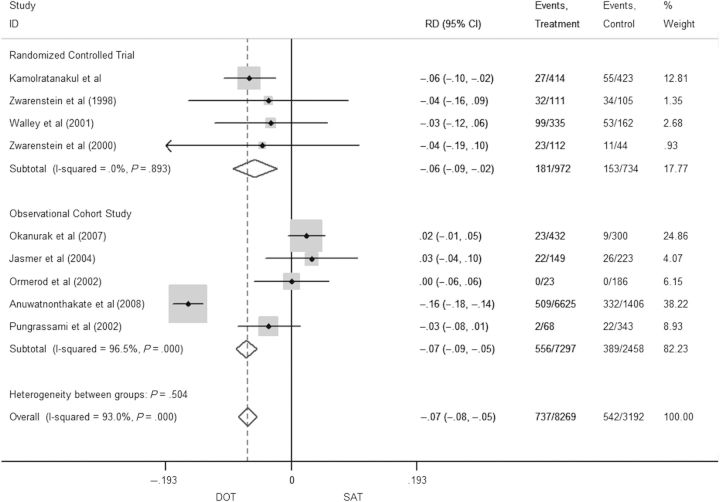
Pooled risk differences for defaulting in patients on directly observed therapy compared to self-administered therapy. Abbreviations: CI, confidence interval; DOT, directly observed therapy; ID, identity; RD, risk difference; SAT, self-administered therapy.

Study [35] reported that 83 (7%) of the 1203 patients assigned to interventions were lost to follow-up, ie, defaulted; however, these defaulters were not reported according to their assigned interventions. Thus, this study was not included in Figure 2.

Abbreviations: CM, community member; CP, continuation phase; DOT, directly observed therapy; FDC, fixed-dose combination; FM, family member; HCW, healthcare worker; IP, intensive phase (first 2 months of tuberculosis therapy); LHW, lay health worker; SAT, self-administered therapy.

^a^ For regimens examined, letters indicate H, isoniazid; R, rifampin; Z, pyrazinamide; E, ethambutol. Subscript denotes number of days per week on therapy and regular script indicates number of months on the regimen.

### DOTS Program Performance

Significant heterogeneity of effect was observed in the 9 of 10 studies that reported defaulting as an outcome (*I^2^* = 68%; *P* = .02); therefore, mixed effects models were employed. Results are shown in Figure 2. SAT (n = 3192) had worse defaulting than DOT (n = 8269), based on pooled RD of −0.05 (95% CI, −.07 to −.04; Figure 2). The pooled IR was 19.4% (95% CI, 18.0%–21.0%) on SAT vs 8.8% (95% CI, 6.1%–9.5%) on DOT (Table 2). If we calculated RR by omitting studies with zero cells, the pooled RR was 0.48 (CI, .43–.54), confirming that whichever one of the 3 effect sizes was utilized, DOT was associated with lower defaulting rates compared to SAT.

**Table 2.** Incidence Rates of Defaulting in Patients on Directly Observed Therapy vs Self-Administered Therapy

Study [Reference]	DOT (95% CI)	Relative Weight (%)	SAT (95% CI)	Relative Weight (%)
Randomized controlled trial				
Kamolratanakul et al [35]	6.5 (4.5–9.3)	25	13.0 (10.1–16.6)	27
Zwarenstein et al [32]	14.4 (9.0–22.2)	24	8.6 (4.5–15.7)	23
Walley et al [5]	32.1 (25.4–39.6)	26	32.7 (25.9–40.3)	27
Zwarenstein et al [33]	20.5 (14.0–29.0)	25	25.0 (14.4–39.7)	23
Pooled IR estimate; REM	16.3 (7.4–32.4)		18.2 (9.4–32.2)	
Heterogeneity measure (*I^2^*)	75%		92%	
Observational cohort				
Okanurak et al [30]	5.3 (3.6–7.9)	28	3.0 (1.6–5.7)	22
Jasmer et al [29]	14.8 (9.9–21.4)	27	11.7 (8.1–16.6)	23
Ormerod et al [27]	2.1 (.1–25.9)	3	.3 (–4.1)	8
Anuwatnonthakate et al [31]	7.7 (7.1–8.4)	35	23.6 (21.5–25.9)	24
Pungrassami et al [28]	2.9 (.7–11.0)	8	6.4 (4.3–9.5)	23
Pooled IR estimate; REM	7.5 (4.9–11.3)		6.8 (2.6–16.5)	
Heterogeneity measure (*I^2^*)	95%		96%	
Overall mixed-effects analysis	8.8 (6.1–9.5)		19.4 (18.0–21.0)	

Abbreviations: CI, confidence interval; DOT, directly observed therapy; IR, incidence rate; REM, random-effects model; SAT, self-administered therapy.

### Effect Size for Microbiologic Outcomes

**Figure 3.**
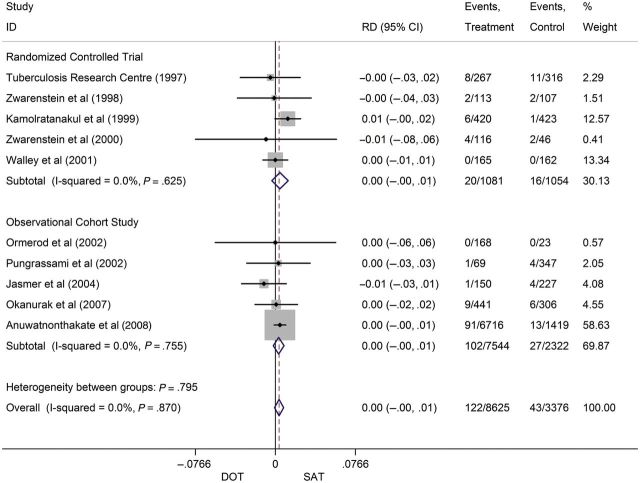
Pooled risk differences for microbiologic failure in patients on directly observed therapy compared to self-administered therapy. Abbreviations: CI, confidence interval; DOT, directly observed therapy; ID, identity; RD, risk difference; SAT, self-administered therapy.

**Figure 4.**
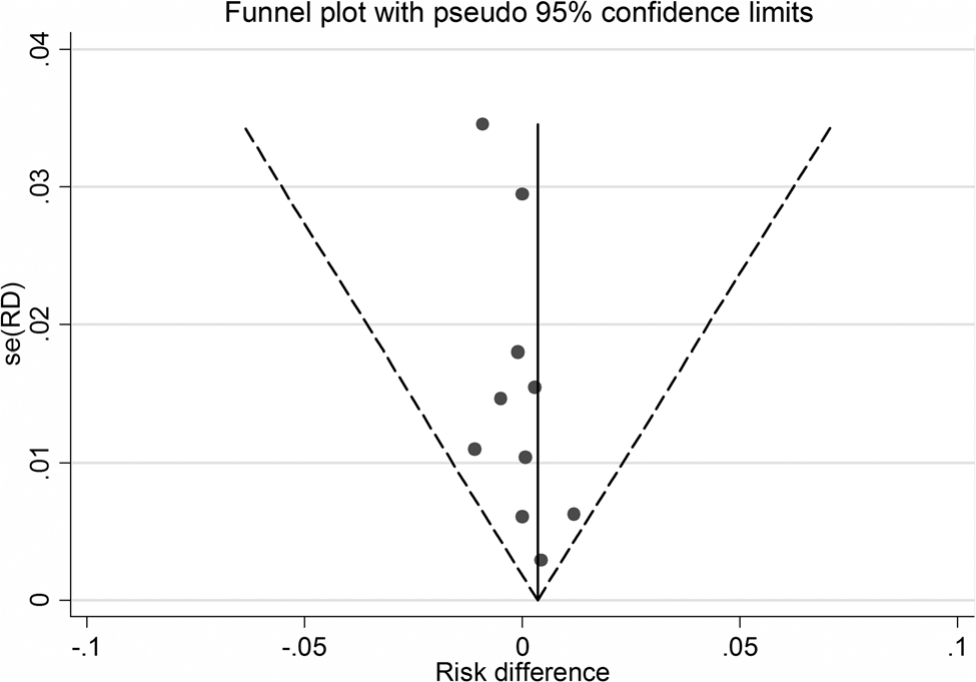
Publication bias analysis and small study effects for microbiologic failure. Abbreviation: RD, risk difference.

For microbiologic failure, 10 studies randomized patients to either SAT (n = 3376) or DOT (n = 8625). The combined *I^2^* was 0%, indicating no significant heterogeneity. Therefore, fixed-effects models were utilized. The pooled RD for patients on DOT vs SAT was 0.0 (CI, <−.01 to .01; Figure 3). The results held true regardless of whether only randomized controlled trials were considered or observational studies were added (Figure 3). No single study demonstrated a significantly higher risk with SAT compared to DOT. The IR was 1.5% (95% CI, 1.3%–1.8%) on DOT vs 1.7% (95% CI, 1.2%–2.2%) on SAT (Table 3). Moreover, the pooled RR for failure on DOT vs SAT was 1.20 (CI, .81–1.78). No significant small study effects or publication bias was observed based on the Egger test and funnel plot examination (Figure 4).

**Table 3.** Incidence Rates of Microbiologic Outcomes in Patients on Directly Observed Therapy vs Self-Administered Therapy

Microbiologic Measures/Study Design	DOT (95% CI)	Relative Weight (%)	SAT (95% CI)	Relative Weight (%)
Microbiologic failure:				
Randomized controlled trial				
Kamolratanakul et al [35]	1.4 (.7–3.2)	29	.2 (–1.7)	15
Zwarenstein et al [32]	1.8 (.5–6.9)	12	1.9 (.5–7.3)	22
Walley et al [5]	.3 (–4.6)	3	.3 (–4.7)	10
Zwarenstein et al [33]	3.6 (1.3–9.1)	21	4.5 (1.1–16.4)	21
Tuberculosis Research Centre [34]	3.1 (1.6–6.1)	35	3.6 (2.0–6.4)	32
Pooled IR estimate; REM	2.2 (1.3–3.7)		1.7 (.6–4.6)	
Heterogeneity measure (*I^2^*)	21%		61%	
Observational cohort				
Okanurak et al [30]	2.1 (1.1–4.0)	9	2.0 (.9–4.4)	22
Jasmer et al [29]	.7 (.1–4.6)	1	1.8 (.7–4.7)	14
Ormerod et al [27]	.3 (–4.5)	1	2.1 (.1–25.9)	2
Anuwatnonthakate et al [31]	1.4 (1.1–1.7)	88	.9 (.5–1.6)	47
Pungrassami et al [28]	1.5 (.2–9.7)	1	1.2 (.4–3.1)	15
Pooled IR estimate	1.4 (1.2–1.7)		1.3 (.9–1.8)	
Heterogeneity measure (*I^2^*)	0%		0%	
Overall mixed-effects analysis	1.5 (1.3–1.8)		1.7 (1.2–2.2)	
Relapse:				
Randomized controlled trial				
Tuberculosis Research Centre [34]	9.3 (6.3–13.7)	100	5.2 (3.1–8.4)	100
Observational cohort				
Jasmer et al [29]	.3 (–5.1)	43	1.9 (.6–5.9)	70
Ormerod et al [27]	4.3 (.6–25.2)	57	.5 (.1–3.7)	30
Pooled IR estimate; REM	1.5(.1–15.7)		1.3 (.4–4.1)	
Heterogeneity measure (*I^2^*)	55%		21%	
Overall mixed-effects analysis	3.7 (.7–17.6)		2.3 (.7–7.2)	
Acquired drug resistance				
Tuberculosis Research Centre [34]	2.7 (1.3–5.6)	71	1.0 (.3–3.0)	60
Jasmer et al [29]	.3 (–5.1)	29	.9 (.2–3.5)	40
Pooled IR estimate; REM	1.5 (.2–9.0)		.9 (.4–2.3)	
Heterogeneity measure (*I^2^*)	52%		0%	
Overall mixed-effects analysis	1.5 (.2–9.0)		.9 (.4–2.3)	

Abbreviations: CI, confidence interval; DOT, directly observed therapy; IR, incidence rate; REM, random-effects model; SAT, self-administered therapy.

**Figure 5.**
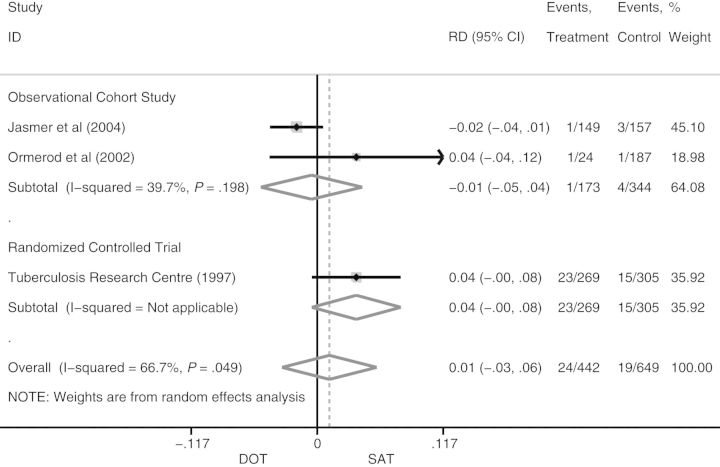
Pooled risk difference for relapse on directly observed therapy compared to self-administered therapy. Abbreviations: CI, confidence interval; DOT, directly observed therapy; ID, identity; RD, risk difference; SAT, self-administered therapy.

**Figure 6.**
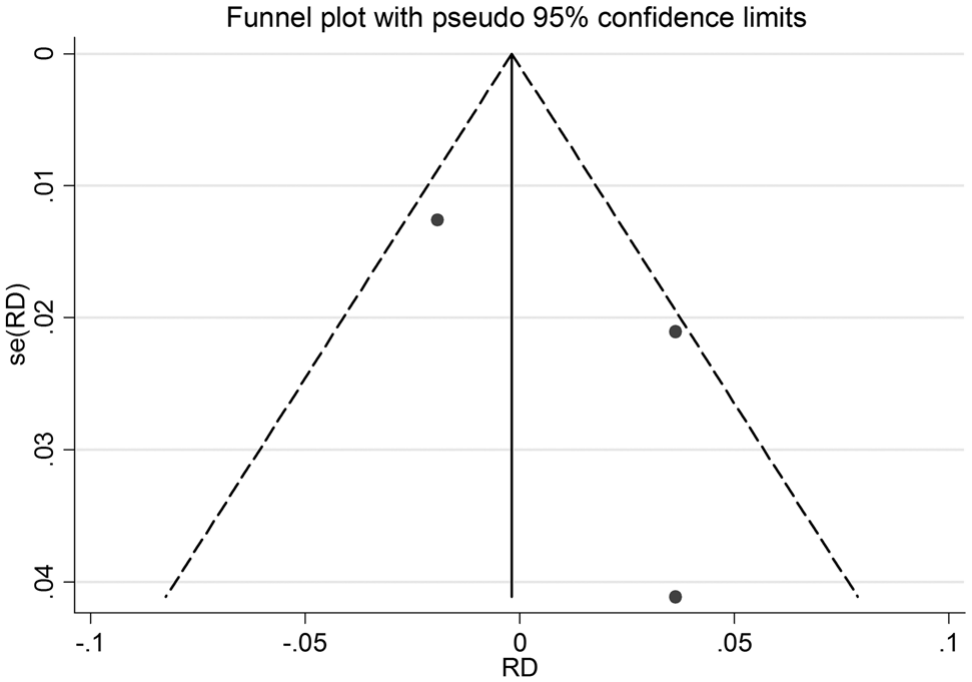
Publication bias analysis and small study effects for relapse. Abbreviation: RD, risk difference.

Three studies reported relapse [27, 29, 34]. The studies had significant heterogeneity (*I^2^* = 68%); therefore, random-effects models were utilized. The pooled RD for relapse on SAT (n = 649) compared to DOT (n = 649) was 0.01 (95% CI, −.03 to .06; Figure 5); the IR was 3.7% (95% CI, .7%–17.6%) on DOT vs 2.3% (95% CI, .7%–7.2%) on SAT (Table 3). The pooled RR was 1.49 (95% CI, 0.31–7.19) for DOT compared to SAT. There was no significant publication bias or small study effects observed (Figure 6).

**Figure 7.**
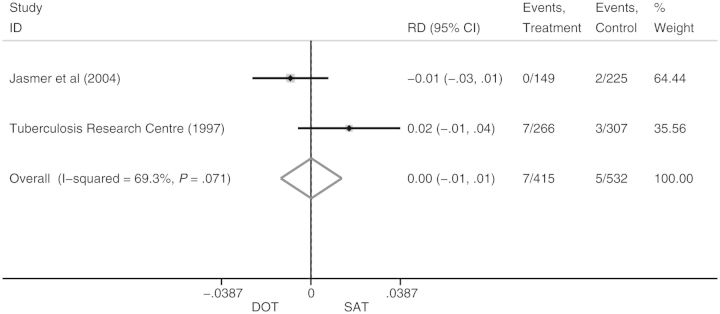
Effect of directly observed therapy vs self-administered therapy on acquired drug resistance. Abbreviations: CI, confidence interval; DOT, directly observed therapy; ID, identity; RD, risk difference; SAT, self-administered therapy.

The 2 ADR studies were heterogeneous (*I^2^* = 69%). The pooled RD was 0.0 (95% CI, −.01 to .01) when DOT (n = 415) was compared to SAT (n = 532; Figure 7); the IR was 1.5% (95% CI, .2%–9.0%) for patients on DOT and 0.9% (95% CI, .40%–2.30%) for patients on SAT (Table 3). The RR of ADR on DOT vs SAT was 1.40 (95% CI, .20–9.98).

### Subgroup and Sensitivity Analysis

In subgroup analysis, microbiologic failure for rural/urban studies was significantly higher on DOT compared to SAT (*P* = .045). The pooled RD for studies performed in urban locales was 0.004 (95% CI, −.016 to .008), whereas the RD from rural/urban studies was 0.004 (95% CI, .00–.009). This suggested that rural patients were more likely to fail on DOT compared to SAT. However, there were no studies performed solely in rural areas. No significant changes in pooled RD were encountered when we systematically removed 1 study at a time in influence analysis (Supplementary Figure 1). Next, we examined whether combining all patients classified as partial DOT with either DOT or SAT, or grouped studies by country (hence program quality), or by study design, led to significant changes in conclusions. There was no significant change in effect size for microbiologic failure or ADR or relapse, for all (Supplementary Figures 2–4).

### Meta-Regression

For microbiologic failure, the percentage residual variation due to heterogeneity for a model comprising study design and study locale was 0% and the joint test for both covariates revealed a *P* = .34. The restricted maximum likelihood estimate for between-study *T^2^* was 0. The RD for study design was 0.01 (95% CI, −.01 to .02), whereas that for study locale was −0.01 (95% CI, −.02 to .01). Thus the findings from the meta-regression demonstrate no other source of variation for the effect obtained, which suggests that there was no significant difference between SAT and DOT.

## DISCUSSION

Well-documented decreases in ADR in several cities and countries have provided strong historical evidence of the success of DOTS, based on decreased defaulting rates [1–4, 8–12]. A prior analysis of Volmink and Garner, in a mixture of patients with latent and active tuberculosis, found that DOT was not superior to SAT for the program-defined outcomes of “completion of treatment” [13]. We found that defaulting rates were indeed reduced by DOT. However, despite the poorer defaulting rates on SAT, we found no difference in microbial failure, ADR, or relapse, between DOT and SAT, similar to our findings in our previously published in vitro hollow-fiber studies [18]. One possible potential explanation for the discrepancies with historical data is that those studies were retrospective, and those that were prospective employed quasi-experimental designs. In evidence-based medicine, the highest quality of scientific evidence comes from >1 properly randomized controlled trial, whereas the lowest quality is generally that of descriptive studies or opinions of authorities, whether or not there is consensus [36]. Notably, no single study demonstrated a significantly higher risk of microbiologic failure with SAT compared to DOT. We speculate that the DOTS program is associated with a large infusion of resources such as upgrade in expertise and a reliable supply of drugs, and that the regular contact with a patient further provides a higher level of support apart from direct supervision of therapy, which would lead to apparent improvement in outcomes in retrospective studies, independent of DOT.

Our findings should not be read as questioning the entire DOTS program, but are limited to supervision of patients swallowing pills. Although the full program is often accompanied by an infusion of resources, the DOT component itself consumes an inordinate portion of that, which is a problem in resource-constrained settings [6]. This may explain the suggested association between rural residence and microbiologic failure. We speculate that economic constraints were the most likely driver accounting for this observation. It may be that requiring patients to frequently come and pick up their medicines or to be observed swallowing their pills could actually impose economic hardships in some parts of the world, leading to microbiologic failure. Moreover, in some high-burden countries, baseline adherence rates measured using validated methods are already >97% on SAT [37], and there may be no room for further improvement in adherence with DOT. We propose that, instead, a concerted effort should be made to shift the resources toward the other possible reasons for such failure, beyond DOTS, including pharmacokinetic/pharmacodynamics and pharmacokinetic and microbial variability [18, 38]. However, the nature of the data reported precluded us from investigating the role of such factors in the current meta-analysis.

There are several limitations to our analyses. First, the WHO definitions we used, particularly for the secondary outcome of “defaulting,” are subject to different interpretations. Second, various DOT supervisors and various forms of DOT were employed by the selected studies, whereas some of the studies did not explicitly state whether DOT was for the initial 2 months of therapy only or for the entire treatment duration. Hence, these data are subject to misclassification bias, which can lead to erroneous failure to reject the null hypothesis [39]. However, the influence and sensitivity analyses we performed did not reveal significant change in the pooled RD, suggesting that these findings are internally robust. Third, it has also been argued that the quality of DOTS programs has an impact on results of meta-analysis, and therefore analysis should be stratified by quality of program. However, we performed a stratified analysis by quality of DOTS program using country as a surrogate, and DOT was still no better than SAT. Fourth, differences in study design and the heterogeneity between studies could make our conclusions less reliable. As an example, it could be that less reliable patients were assigned to DOT whereas more reliable patients were assigned SAT in the observational studies, which would bias the results. However, analysis of randomized studies alone vs analysis that included observational studies did not alter the conclusions (Figure 3). Fifth, ADR and relapse studies were fewer and these were of different study design. The single randomized clinical trial revealed higher risk for relapse with SAT compared to DOT when RR was calculated (RR, 1.74 [95% CI, .93–3.26]); however, it did not achieve statistical significance. For the observational studies, the pooled RR was 1.13 (95% CI, .02–54.91). These results were partly due to zero cells and the imputation strategies inherent with using RR as effect size. That is why our primary effect sizes were RD and IR, which require no such imputation. The differences by study design vanished when those effect sizes were employed. Finally, an inherent limitation of meta-analyses is that some influential studies may be missed during the search, thereby biasing the studies. However, we excluded no studies by publication language, examined the Cochrane database and the gray literature, and performed a manual search of references in key publications, in order to minimize bias.

In conclusion, our evidence-based medicine approach found that DOT was not superior to SAT in terms of microbiologic outcomes. Other causes of poor microbiologic outcomes should be sought in new studies.

## Supplementary Data

Supplementary materials are available at *Clinical Infectious Diseases* online (http://cid.oxfordjournals.org/). Supplementary materials consist of data provided by the author that are published to benefit the reader. The posted materials are not copyedited. The contents of all supplementary data are the sole responsibility of the authors. Questions or messages regarding errors should be addressed to the author.

Supplementary Data
