# Supplementary material for: A Meta-Analysis of Self-Administered vs Directly Observed Therapy Effect on Microbiologic Failure, Relapse, and Acquired Drug Resistance in Tuberculosis Patients
Source: Clin Infect Dis. 2013 Mar 13;57(1):21–31. doi: 10.1093/cid/cit167 (PMC3669525; doi:10.1093/cid/cit167)
Supplement: Supplementary Data [file supp_57_1_21__index.html]

A Meta-Analysis of Self-Administered vs Directly Observed Therapy Effect on Microbiologic Failure, Relapse, and Acquired Drug Resistance in Tuberculosis Patients — A Meta-Analysis of Self-Administered vs Directly Observed Therapy Effect on Microbiologic Failure, Relapse, and Acquired Drug Resistance in Tuberculosis Patients — Supplementary Data 

# A Meta-Analysis of Self-Administered vs Directly Observed Therapy Effect on Microbiologic Failure, Relapse, and Acquired Drug Resistance in Tuberculosis Patients

## Supplementary Data

Supplementary Data

**Files in this Data Supplement:**

- Supplementary Data - Doc file
- Supplementary Figure 1 - tif file
- Supplementary Figure 2 - eps file
- Supplementary Figure 3 - eps file
- Supplementary Figure 4 - eps file
