# Supplementary material for: A Meta-Analysis of Self-Administered vs Directly Observed Therapy Effect on Microbiologic Failure, Relapse, and Acquired Drug Resistance in Tuberculosis Patients
Source: Clin Infect Dis. 2013 Mar 13;57(1):21–31. doi: 10.1093/cid/cit167 (PMC3669525; doi:10.1093/cid/cit167)
Supplement: Supplementary Data [file supp_cit167_cit167supp.doc]

**SUPPLEMENTARY FIGURE LEGENDS**.

**Supplementary Figure 1.**  **Single study influence analysis.**

**Supplementary Figure 2. Effect of country where study conducted.** This measure was used as a surrogate for quality of the directly observed therapy program, as opposed to quality of study.

**Supplementary Figure 3. Effect of combining partial-DOT with DOT.**

**Supplementary Figure 4. Effect of combining partial-DOT with SAT.**
