# Supplementary figures and images for: A Meta-Analysis of Self-Administered vs Directly Observed Therapy Effect on Microbiologic Failure, Relapse, and Acquired Drug Resistance in Tuberculosis Patients
Source: Clin Infect Dis. 2013 Mar 13;57(1):21–31. doi: 10.1093/cid/cit167 (PMC3669525; doi:10.1093/cid/cit167)

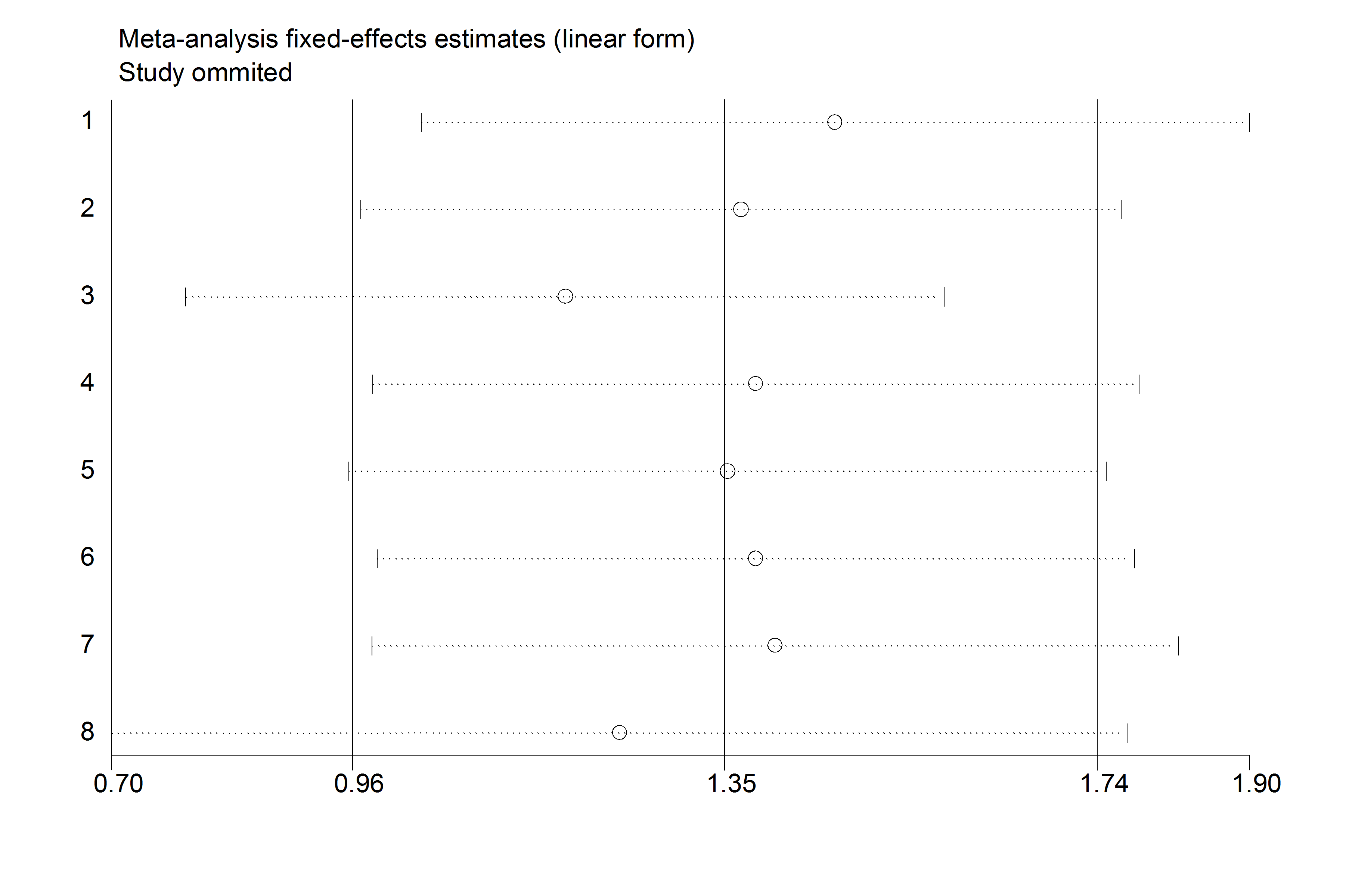

Supplement: Supplementary Data [file supp_cit167_cit167supp_fig1.tif]
